# Heat Shock-Induced Accumulation of the Glycogen Synthase Kinase 3-Like Kinase BRASSINOSTEROID INSENSITIVE 2 Promotes Early Flowering but Reduces Thermotolerance in *Arabidopsis*

**DOI:** 10.3389/fpls.2022.838062

**Published:** 2022-01-27

**Authors:** Huimin Ren, Xuedan Wu, Weishuang Zhao, Yuetian Wang, Daye Sun, Kang Gao, Wenqiang Tang

**Affiliations:** Ministry of Education Key Laboratory of Molecular and Cellular Biology, Hebei Collaboration Innovation Center for Cell Signaling, Hebei Key Laboratory of Molecular and Cellular Biology, College of Life Sciences, Hebei Normal University, Shijiazhuang, China

**Keywords:** heat shock, brassinosteroid, BIN2, ABI5, thermotolerance, flowering

## Abstract

Brassinosteroids (BRs) are essential plant growth- and development-regulating phytohormones. When applied exogenously, BRs ameliorate heat shock (HS)-induced cell damage and enhance plant thermotolerance; however, the molecular mechanism by which BRs regulate plant thermotolerance is unknown. In this study, by analyzing the thermotolerance of a series of BR signaling mutants and plants that overexpressed different BR signaling components, we obtained comprehensive data showing that BRASSINOSTEROID INSENSITIVE 2 (BIN2) plays a major role in mediating the crosstalk between BR signaling and plant HS responses. By RNA-Seq, 608 HS- and BIN2-regulated genes were identified. An analysis of the 1-kb promoter sequences of these genes showed enrichment of an abscisic acid (ABA) INSENSITIVE 5 (ABI5)-binding *cis*-element. Physiological studies showed that thermotolerance was reduced in *bin2-1* mutant and *ABI5-OX* plants but increased in the *abi5* mutant, and that the *abi5* mutation could recover the thermotolerance of *bin2-1* plants to a wild-type level, suggesting that ABI5 functions downstream of BIN2 in regulating plant thermotolerance. Further, HS treatment increased the cellular abundance of BIN2. Both *bin2-1* mutant and *BIN2-OX* plants showed early flowering, while the *BIN2* loss-of-function mutant *bin2-3 bil1 bil2* flowered late. Given these findings, we propose that under HS conditions plants increase BIN2 activity to promote early flowering and ensure species survival; however, this reduces the thermotolerance and survivability of individual plants partially by activating ABI5.

## Introduction

With the increasing impact of the greenhouse effect, it is estimated that the global surface temperature has increased by approximately 1.09°C compared with that observed in the pre-industrial era^1^. Because of global warming, the appearance of seasonally extreme heat has become increasingly frequent. This makes heat stress one of the most important environmental factors limiting plant growth and productivity. Heat stress has a deleterious influence on almost every aspect of plant growth and development; it can change the thylakoid structure in chloroplasts, inactivate Rubisco, reduce the number of photosynthetic pigments, and destroy photosystem II to quickly inhibit photosynthesis (Danilova et al., 2018; Dogra et al., 2019; Hu et al., 2020). Heat stress also inhibits plant development, promotes early flowering, causes tissue and organ senescence, inhibits pollen formation and fertilization, reduces seed vigor, and suppresses seed germination and grain filling (Ma et al., 2019; Jung et al., 2020; Li et al., 2021a; Poidevin et al., 2021; Ren et al., 2021). One study showed that from 1964 to 2007, drought and extreme heat significantly reduced global cereal production by 9–10% (Lesk et al., 2016). Therefore, uncovering the mechanism by which plants respond to heat stress and using that knowledge to promote the breeding of heat stress-tolerant crops will protect future global agricultural productivity.

When they encounter high temperatures, plants quickly reprogram their cellular transcriptome and metabolism to increase the production and accumulation of heat shock (HS) proteins (HSPs), antioxidants, and osmolytes to enhance thermotolerance (Li et al., 2019; Ali et al., 2020). HS also regulates the expression of phytohormone biosynthesis and hormone response-related genes (Li et al., 2019, 2021b). Phytohormones such as abscisic acid (ABA), cytokinin, salicylic acid (SA), and jasmonic acid (JA) could all enhance the HS tolerance of plants (Li et al., 2021b); however, it is unclear whether these phytohormones function independently or interdependently to ameliorate HS-induced damage and increase plant heat tolerance.

Brassinosteroids (BRs) are a group of plant steroid hormones. Since their discovery in the 1970s, BRs have been shown to play essential roles in regulating diverse growth and developmental processes in plants (Yang et al., 2011). Genetic, biochemical, and molecular biological studies have revealed the signaling pathway by which BRs regulate plant growth and development. Extracellular BRs are perceived by the plasma membrane-localized receptor BRASSINOSTEROID INSENSITIVE 1 (BRI1) and its co-receptor, BRI1 ASSOCIATED RECEPTOR KINASE 1 (BAK1) (Li and Chory, 1997; Li et al., 2002; Nam and Li, 2002). BR binding promotes transphosphorylation between BRI1 and BAK1, and it activates downstream signaling components, including BR SIGNALING KINASEs (BSKs), CONSTITUTIVE DIFFERENTIAL GROWTH 1 (CDG1), and BRI1-SUPPRESSOR 1 (BSU1), via sequential protein phosphorylation (Mora-Garcia et al., 2004; Tang et al., 2008; Kim et al., 2009, 2011). Upon being activated by BR signaling, the protein phosphatase BSU1 dephosphorylates and inactivates downstream BRASSINOSTEROID INSENSITIVE 2 (BIN2) family protein kinases, and it promotes BIN2 degradation via the KINK SUPPRESSED in *bzr1-1D* (KIB1)-mediated proteasome pathway (Peng et al., 2008; Kim et al., 2009; Zhu et al., 2017). BIN2 is a negative regulator of BR signaling. When the extracellular BR level is low, BIN2 phosphorylation inactivates two homologous transcription factors, BRASSINAZOLE RESISTANT 1 (BZR1) and BRI1-EMS-SUPPRESSOR 1 (BES1), resulting in the accumulation of BZR1/BES1 in the cytoplasm (Vert and Chory, 2006; Gampala et al., 2007). BR signaling inactivates BIN2 and promotes the translocation of phosphorylated BZR1 from the cytoplasm to the nucleus where it is dephosphorylated by the protein phosphatase PP2A; it then binds to the promoters of downstream target genes, regulating their expression (Sun et al., 2010; Tang et al., 2011; Wang et al., 2021).

Besides its role in regulating plant growth and development, BR signaling has been found to regulate plant adaptations to various environmental abiotic stresses such as high or low temperatures, drought, and salt (Planas-Riverola et al., 2019). When applied exogenously, BRs increase the thermotolerance of a variety of plant species, including *Arabidopsis*, rice, tomato, barley, eggplant, *Cucumis melo*, and *Brassica napus* (Kagale et al., 2007; Sadura and Janeczko, 2018; Yin et al., 2018). Consistent with these results, mutants deficient in BR biosynthesis or signaling are more susceptible to HS treatment (Yin et al., 2018; Fang et al., 2020), while plants overexpressing BR biosynthesis- or signaling-related genes show increased HS tolerance (Sahni et al., 2016; Yin et al., 2018). Meanwhile, reports indicate that BR biosynthesis- or signaling-deficient mutants have greater HS tolerance than wild-type plants (Mazorra et al., 2011; Sadura et al., 2019). The contradictory HS-related phenotypes observed in these studies suggest that BRs regulate HS responses in plants in a tissue/organ-specific manner and via multiple mechanisms. Therefore, identifying the components that mediate the crosstalk between BR signaling and plant HS responses is necessary to uncover the mechanism by which BRs regulate plant thermotolerance.

In this study, we systematically analyzed the thermotolerance of several BR biosynthesis/signaling-deficient or hyperactive *Arabidopsis* mutants and transgenic plants. Our data show that BIN2 plays an essential role in mediating the regulation of plant HS responses by BR signaling. HS induced the accumulation of BIN2, which accelerated the life cycle of *Arabidopsis* plants under HS conditions by promoting early flowering, while decreasing their thermotolerance. These results reveal a novel mechanism by which plants promote early flowering to adapt to HS under natural conditions, and they explain why BR treatment increases the thermotolerance of *Arabidopsis* plants.

## Materials and Methods

### Plant Materials and Growth Conditions

Most of the *Arabidopsis* mutants and transgenic plants used in this study were reported previously (Wang et al., 2002; Mora-Garcia et al., 2004; Tang et al., 2008; Yan et al., 2009; Hu and Yu, 2014; Zhou et al., 2015; Yang et al., 2016; Chen et al., 2019). Seeds (30 per genotype) were surface-sterilized and sown on glass plates containing 30 ml of half-strength Murashige and Skoog (1/2 MS) agar medium (0.6% Phytagel, 0.5 × MS basal salt mixture, and 1% sucrose, with the pH adjusted to 5.7–5.8 using KOH). For BR treatment, Petri dishes with two compartments were used; each compartment contained 15 ml of 1/2 MS agar medium supplied with or without 0.1 μM epibrassinolide (eBL). The seeds were stratified at 4°C for 2–3 days then placed in a growth chamber (Percival Scientific, Perry, IA, United States) under long-day (LD; 16 h of light/8 h of dark) conditions at 22°C for 7 days before HS treatment, material harvesting, or transplantation to soil with subsequent growth in a greenhouse under the same growth conditions in order to examine flowering-related phenotypes.

### Thermotolerance Assays

For the basal thermotolerance (BT) assay, plates containing 7-day-old seedlings were transferred to a new growth chamber (Percival Scientific) with the inside temperature preset to 45°C (HS treatment) or kept in the same growth chamber (22°C; control) and left for 60–75 min. For the acclimated thermotolerance (AT) assay, plates containing 7-day-old seedlings were first exposed to 37°C for 1 h. The plants were then allowed to recover at 22°C for 2 h before being subjected to 45°C or 22°C in a growth chamber (Percival Scientific) for 3 h. In both the BT and AT assays, after HS treatment the plants were allowed to continue to grow at 22°C under LD conditions for 3–4 days before being photographed and examined for survival. Each HS treated glass plate is considered as one biological replicate. In general, at least 30 biological replicates collected from four to five independent experiments were used for survival rate quantification, unless otherwise indicated.

### Ion Leakage Assay

The electrical conductivity of deionized water was set as C0. Immediately after HS treatment, plants were incubated in deionized water (10 ml per 30 seedlings) and shaken mildly at room temperature for 1 h after which the conductivity of the solution was measured (C1). The seedlings were then boiled in the same deionized water for 1 h and the conductivity of the solution was measured again after cooling to room temperature (C2). Ion leakage (%) was calculated as the ratio of (C1 − C0)/(C2 − C0) (Li et al., 2017).

### RNA Sequencing and Bioinformatic Analysis

Seven-day-old seedlings grown on 1/2 MS agar medium were transferred to a 37°C growth chamber (Percival Scientific) or kept in the same 22°C growth chamber (Percival Scientific) and left for 1 h before tissue samples were harvested for total RNA extraction using an RNeasy Plant Mini Kit (Qiagen, Germantown, MD, United States). The 2 μg of RNA per sample was sent for Oxford Nanopore Technologies-based single-molecule real-time sequencing following a standard protocol. Super-long reads, which contain sequence information about a single complete transcript, were identified and aligned to the *Arabidopsis* reference genome (TAIR11) using bioinformatics analysis tools (Minimap2) on the BMKCloud platform^2^. Three biological replicates were used, and differentially expressed genes (DEGs) were identified using the edgeR package (version 3.8.6) with the following parameters: fold change (FC) ≥ 2 and *p*-value < 0.05.

Gene Ontology (GO) analysis was performed online using AgriGO^3^. For *cis*-element motif enrichment analysis, promoter and 5′-untranslated sequences (−1000 to −1) of the target genes were downloaded from the Arabidopsis Resource Center^4^ (TAIR11) and loaded onto the MEME Suite webtool (version 5.4.1) for motif-based sequence analysis^5^.

### Gene Expression Analysis

One-week-old seedlings grown on 1/2 MS agar medium were treated at 37°C or 22°C in growth chambers for the indicated time. Harvested plant tissue was ground in liquid nitrogen to a fine powder, and total RNA was extracted from 100 mg of tissue using TRIzol Reagent (CWBio, Taizhou, Jiangsu, China) according to the manual provided by the manufacturer. First-strand cDNA was synthesized from 1 μg of total RNA using HiScript II Q Select RT SuperMix for qPCR (+gDNA wiper) (Vazyme Biotech Co., Nanjing, Jiangsu, China). Quantitative PCR (qPCR) was performed using ChamQ Universal SYBR qPCR Master Mix (Vazyme Biotech Co.) on a C1000 Touch Thermal Cycler CFX384 (Bio-Rad Hercules, CA, United States). The relative abundance of each transcript was determined by the comparative threshold cycle method (Schmittgen and Livak, 2008), using *UBC30* as an equal loading control. The gene-specific primers used are listed in Supplementary Table 4.

### Immunoblotting

Plant tissues were ground to a fine powder in liquid nitrogen. In a heat block, SDS sample buffer (0.125 M Tris-HCl, pH 6.8, 4% SDS, 20% glycerol, 2% β-mercaptoethanol, bromophenol blue, and 1× protease inhibitor cocktail) was preheated to 95°C and then added to the tissue powder at a ratio of 20 μl per 10 mg of tissue powder. After vigorous vortexing, the homogenate was heated at 95°C for 5 min and then centrifuged at 12,000 × *g* for 10 min at 4°C. The supernatant was used for SDS-PAGE and immunoblotting with anti-GFP (Roche, Basel, Switzerland) or anti-tubulin (Sigma-Aldrich, St. Louis, MO, United States) antibodies.

## Results

### Brassinosteroids Regulate Basal Thermotolerance and Acclimated Thermotolerance in Plants

**FIGURE 1:**
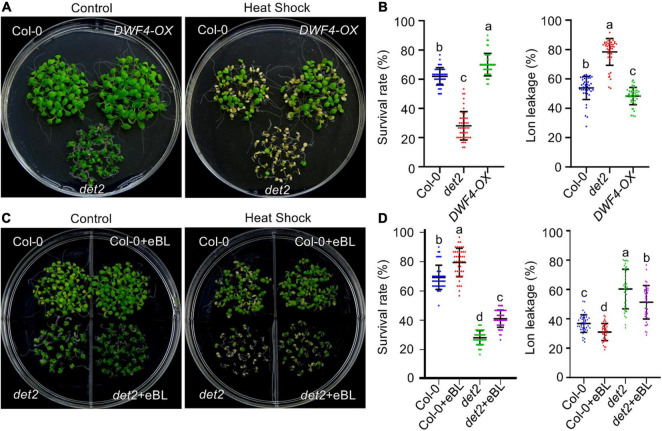
Brassinosteroid regulates acquired thermotolerance in *Arabidopsis*. **(A,C)** The phenotypes of HS-treated and untreated plants. One-week-old seedlings grown on 1/2 MS agar medium supplemented with or without 0.1 μM eBL under LD conditions at 22°C were exposed to 37°C for 1 h. After recovering at 22°C for 2 h, the seedlings were exposed to 22°C (control) or 45°C (HS) for 3 h and then allowed to recover at 22°C for 3 days before being imaged. **(B,D)** Quantification of the survival rates of and ion leakage from the plants shown in panels **(A,C)**. Error bars indicate the mean ± standard deviation (SD). Statistically significant differences are indicated by different lowercase letters (*p* < 0.05, one-way ANOVA).

To determine the role of BRs in regulating plant HS tolerance, we first evaluated whether altering the endogenous BR biosynthesis level would alter the thermotolerance of *Arabidopsis* plants. Compared with wild-type plants, the BT and AT were significantly reduced in the BR biosynthesis-deficient mutant *det2*, but increased in transgenic plants overexpressing the rate-limiting BR biosynthesis gene *DWF4* (*DWF4-OX*) (Supplementary Figure 1A and Figures 1A,B). Electrolyte leakage is an indicator of HS-induced plasma membrane damage. Corresponding with our thermotolerance result, after HS treatment electrolyte leakage was significantly increased in the *det2* mutant but reduced in *DWF4-OX* plants (Figure 1B).

Next, we tested whether exogenously applied BRs would recover the reduced thermotolerance observed in the *det2* mutant. Both wild-type and *det2* mutant plants were grown in two compartment plates containing 1/2 MS agar medium supplied with or without 0.1 μM eBL for 1 week before high temperature treatment. The addition of eBL to the growth medium significantly improved the BT and AT, and reduced electrolyte leakage, in wild-type and *det2* plants (Supplementary Figure 1B and Figures 1C,D). Nevertheless, *det2* mutants grown in a eBL-containing medium were much more sensitive to HS compared with wild-type seedlings grown in the absence of BR. These results show that although exogenously applied eBL could enhance plant thermotolerance, they did not fully complement the reduced thermotolerance of the *det2* mutant. Thus, the spatial-temporal activation of BR signaling *in vivo* plays a critical role in regulating plant responses to high temperatures.

### BRASSINOSTEROID INSENSITIVE 1, BRASSINOSTEROID SIGNALING KINASE 3, and BRASSINOSTEROID INSENSITIVE 1-SUPPRESSOR 1 Contribute to Brassinosteroid-Regulated Thermotolerance in Plants

**FIGURE 2:**
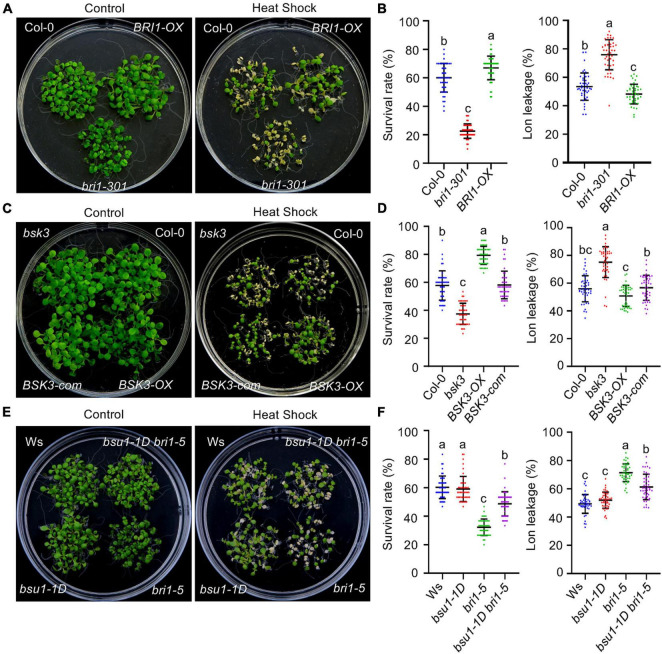
The BR signaling components BRI1, BSK3, and BSU1 regulate acquired thermotolerance in *Arabidopsis*. **(A,C,E)** Photographs of HS-treated and untreated plants after 3 days of recovery at 22°C. **(B,D,F)** Quantification of the survival rates of and ion leakage from the plants shown in panels **(A,C,E)**. Error bars indicate the mean ± SD. Statistically significant differences are indicated by different lowercase letters (*p* < 0.05, one-way ANOVA).

To investigate which BR signaling component plays a major role in mediating BR-enhanced HS tolerance in plants, the survival rates of BR signaling mutants and transgenic plants overexpressing BR signaling components were systematically assessed following HS treatment. Because strong BR signaling mutants show extreme dwarfism, weak BR signaling mutants were used to avoid potential artifacts caused by the severe vegetative growth retardation observed in strong BR signaling mutants. Both the BT and AT were significantly reduced in the BR receptor (BRI1)-deficient mutants *bri1-301* and *bri1-5* (Figures 2A,B,E,F and Supplementary Figures 1C,E), as well as in the mutant *bsk3* (Figures 2C,D and Supplementary Figure 1D), but increased in transgenic plants overexpressing BRI1 (Figures 2A,B and Supplementary Figure 1C) and BSK3 (Figures 2C,D and Supplementary Figure 1D). The reduced thermotolerance of the *bsk3* mutant is caused by the knock down of the expression of *BSK3* (Tang et al., 2008); introducing the BSK3 genomic sequence fused with a C-terminal YFP tag into the *bsk3* mutant (BSK3-com) fully recovered the thermotolerance of the *bsk3* mutant to a wild-type level (Figures 2C,D and Supplementary Figure 1D). *bsu1-1D* is a gain-of-function activation-tagged mutant that overexpresses BSU1. Similar to its ability to recover the vegetative growth of the *bri1-5* mutant (Mora-Garcia et al., 2004), *bsu1-1D* partially restored the reduced thermotolerance of *bri1-5* plants, even though the BT and AT of *bsu1-1D* were similar to those of the wild-type control (Figures 2E,F and Supplementary Figure 1E). An analysis of electrolyte leakage in these HS-treated plants supported our altered thermotolerance data (Figures 2B,D,F). Together, these results suggest that BRI1-, BSK3-, and BSU1-mediated BR signaling contributes to the enhanced thermotolerance of BR-treated plants.

### BRASSINOSTEROID INSENSITIVE 2 Mediates Crosstalk Between the Brassinosteroid Signaling Pathway and Plant Thermotolerance

**FIGURE 3:**
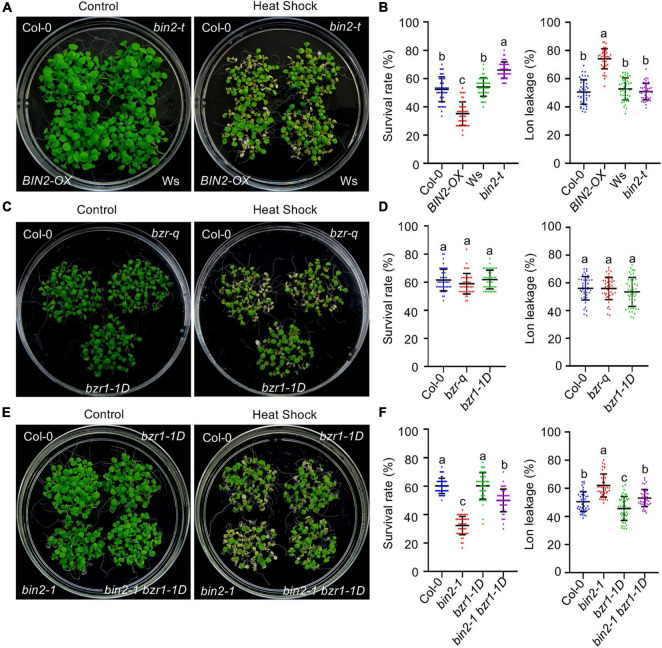
The BR signaling component BIN2 (but not BZRs) plays a major role in regulating acquired thermotolerance in *Arabidopsis*. **(A,C,E)** Photographs of HS-treated and untreated plants after 3 days of recovery at 22°C. *bin2-t* represents the *bin2-3 bil1 bil2* triple mutant and *bzr-q* is a *bes1 beh1 beh2 beh3* quadruple mutant. **(B,D,F)** Quantification of the survival rates of and ion leakage from the plants shown in panels **(A,C,E)**. Error bars indicate the mean ± SD. Different lowercase letters indicate statistically significant differences (*p* < 0.05, one-way ANOVA).

BRASSINOSTEROID INSENSITIVE 2 is the downstream target of BSU1 in BR signaling. BIN2 and its homologs BIN2-LIKE1 (BIL1) and BIL2 function redundantly in regulating BR signaling (Yan et al., 2009). To explore the relationship between BIN2 and BR-regulated plant HS responses, the thermotolerance of the loss-of-function mutant *bin2-3 bil1 bil2* (*bin2-t*) and *35Spro:BIN2-myc* (*BIN2-OX*) plants were examined. When exposed to HS, both the BT and AT were significantly increased in the *bin2-t* mutant but decreased in *BIN2-OX* plants (Figures 3A,B and Supplementary Figure 1F). Correspondingly, electrolyte leakage was increased in the *BIN2-OX* plants (Figure 3B).

BRASSINAZOLE RESISTANT 1/BRI1-EMS-SUPPRESSOR 1 play an essential role in BR-regulated gene expression, and the activity of BZR1/BES1 in BR signaling is tightly controlled by BIN2 phosphorylation (Vert and Chory, 2006). Besides *BZR1* and *BES1*, there are four additional *BZR1/BES1 HOMOLOG* (*BEH*) genes encoded in the *Arabidopsis* genome. These BZR1/BES1 family transcription factors (BZRs) play redundant regulatory roles in BR signaling (Chen et al., 2019). We therefore tested whether there is a correlation between cellular BZR activity and plant thermotolerance. Upon HS treatment, *bes1 beh1 beh2 beh3* quadruple mutant (*bzr-q*) plants (Chen et al., 2019) and *bzr1-1D*, a gain-of-function *bzr1* mutant with constitutively activated expression of downstream BR target genes even in the absence of BRs (Wang et al., 2002), exhibited similar survival rates and electrolyte leakage compared with wild-type plants under both non-acclimated and acclimated conditions (Figures 3C,D and Supplementary Figure 1G), indicating BZRs do not play a major role in regulating plant thermotolerance.

To obtain additional information on the role of BZRs in regulating plant HS responses, the thermotolerance of *bin2-1*, a gain-of-function mutant that stabilizes BIN2 (Li and Nam, 2002), and *bin2-1 bzr1-1D* mutant plants was examined. Similar to *BIN2-OX* plants, the BT and AT were significantly decreased and electrolyte leakage after HS was increased in the *bin2-1* mutant. Introducing the *bzr1-1D* mutation into *bin2-1* mutant plants increased the survival rate from 37.7 ± 6.3% to 52.7 ± 6.2% under non-acclimated conditions, and from 32.3 ± 6.4% to 49.9 ± 8% under acclimated conditions. However, when compared with the survival rate of wild-type control plants (68.3 ± 7.4% under BT conditions and 60.1 ± 5.4% under AT conditions), the *bin2-1 bzr1-1D* double mutant was still hypersensitive to HS (Figures 3E,F and Supplementary Figure 1H). Consistent with these survival data, plant electrolyte leakage after HS was significantly increased in the *bin2-1* mutant, and partially recovered but still lower than in the wild-type control in the *bin2-1 bzr1-1D* double mutant (Figure 3F). Together, these results suggest that BIN2 plays a dominant role in mediating crosstalk between the BR signaling pathway and plant HS responses. However, the enhanced thermotolerance observed in the *bin2-1 bzr1-1D* mutant (compared with *bin2-1*) suggests that BIN2 and BZR1 oppositely regulate the activity of a subset of common downstream targets to influence plant thermotolerance.

### Heat Shock Increases BRASSINOSTEROID INSENSITIVE 2 Protein Abundance and the Nuclear Accumulation of BRASSINAZOLE RESISTANT 1

**FIGURE 4:**
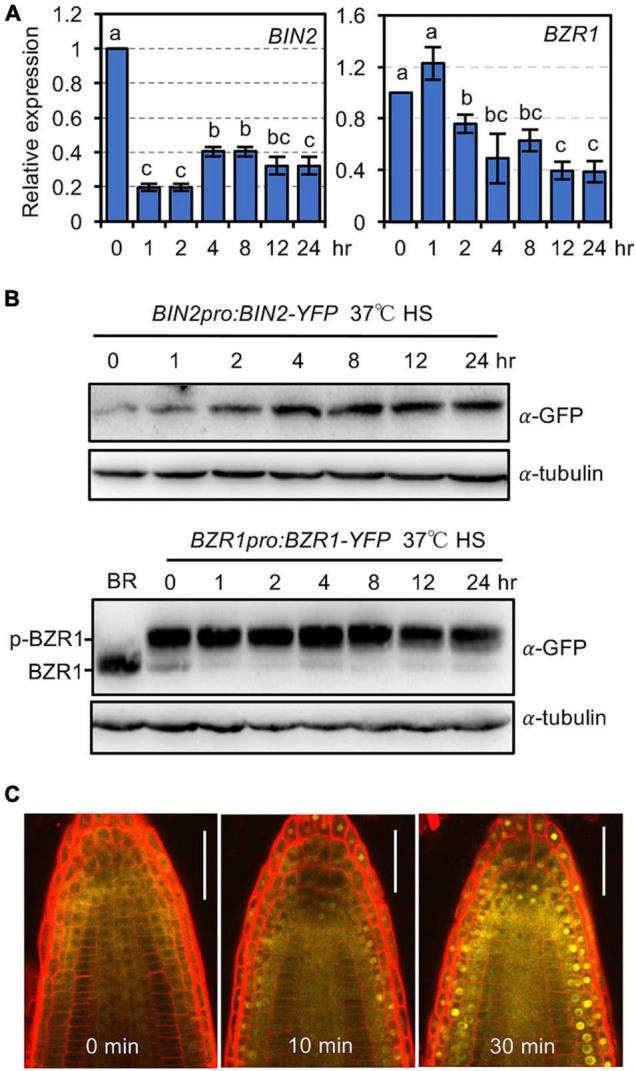
Heat shock induces BIN2 protein accumulation and promotes the nuclear localization of BZR1. **(A)** The relative expression of *BIN2* and *BZR1* transcripts in 1-week-old Col-0 seedlings grown at 37°C for the indicated time. Error bars indicate the mean ± SD (*n* = 3). Statistically significant differences are indicated by different lowercase letters (*p* < 0.05, one-way ANOVA). **(B)** Immunoblotting revealed the abundance of BIN2-YFP and BZR1-YFP in 1-week-old seedlings treated at 37°C for the indicated time. For BR treatment, seedlings were soaked in ddH_2_O containing 1 μM eBL for 2 h. **(C)** Confocal microscopic examination of the subcellular localization of BZR1-YFP in the roots of 5-day-old plants grown on 1/2 MS agar medium containing 0.5 μM PCZ. The plates were treated at 37°C for the indicated time, and then the roots were mounted in a solution containing 0.5 μM PCZ and 1 mg/mL of propidium iodide and observed. Scale bars, 20 μm.

The above results suggested that BR signaling inactivates BIN2 to increase plant thermotolerance. The question is why plants would need to activate BR signaling to defend themselves against HS damage under natural conditions. Would HS alter the activity and/or subcellular localization of BIN2 and BZRs? By real-time quantitative PCR (qPCR), we found that treating plants at 37°C for 1 or 2 h significantly inhibited the expression of *BIN2* and *BZR1*, respectively, and that the transcript levels of *BIN2* and *BZR1* continued to be suppressed by treatment at 37°C for up to 24 h (Figure 4A). We also examined whether HS affected the protein abundance of BIN2 and BZR1 using *BIN2pro:BIN2-YFP* and *BZR1pro:BZR1-YFP* transgenic plants. In contrast to our transcription data, treatment at 37°C for 1 h increased the BIN2-YFP protein level, and the abundance of BIN2-YFP continued to increase as the HS treatment period was increased (Figure 4B). In comparison, the protein abundance of phosphorylated BZR1-YFP was not obviously altered, while the level of dephosphorylated BZR1 was significantly reduced at 1 h after HS treatment (Figure 4B).

Brassinosteroid signaling promotes the nuclear accumulation and dephosphorylation of BZR1 to activate downstream target genes. Previously, it was shown that ambient high temperature treatment induced the nuclear localization of BZR1 (Ibañez et al., 2018). To investigate whether HS also promotes the nuclear accumulation of BZR1, the subcellular localization of BZR1-YFP was examined in 1-week-old root cells from *BZR1pro:BZR1-YFP*-expressing plants by confocal microscopy. Nuclear localization of BZR1-YFP was observed after 10 min of treatment at 37°C, even in the presence of 0.5 μM propiconazole (PCZ), a BR biosynthesis inhibitor (Figure 4C). The nuclear localization of BZR1-YFP became increasingly obvious when the HS treatment period was extended to 30 min (Figure 4C). Considering that HS increased the cellular BIN2 protein level but decreased the dephosphorylated BZR1-YFP level, it is likely that the HS-induced accumulation of BIN2 prevented nuclear-localized BZR1 from being dephosphorylated by PP2A.

### The Identification of BRASSINOSTEROID INSENSITIVE 2 and Heat Shock Coregulated Genes

**FIGURE 5:**
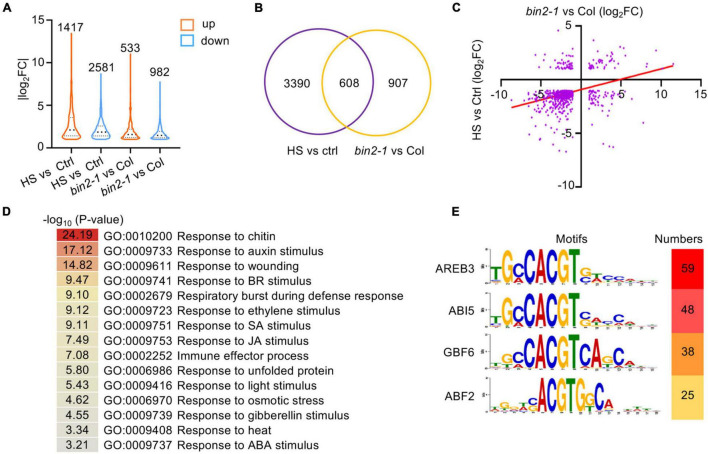
Transcriptome analysis of HS- and BIN2-regulated genes. **(A)** The numbers and distribution of HS- and BIN2-regulated genes. One-week-old seedlings grown on 1/2 MS agar medium were treated with (Col-0) or without (*bin2-1* and Col-0) HS at 37°C for 1 h. **(B)** A Venn diagram showing the number of genes that were differentially regulated by HS treatment or the *bin2-1* mutation. **(C)** The relative expression levels of the 608 HS and BIN2 coregulated genes shown in panel **(B)**. *X*-axis: the relative expression levels determined by comparing HS-treated and untreated Col-0 seedlings. Y-axis: the relative expression levels determined by comparing *bin2-1* and Col-0 plants grown at 22°C. **(D)** GO classification of the 608 HS and BIN2 coregulated genes. **(E)** The top four enriched *cis*-elements in the 608 HS and BIN2 coregulated genes are shown.

To gain insight into BIN2-mediated crosstalk between the BR signaling pathway and plant HS responses, RNA-Seq was employed to identify BIN2 and HS coregulated genes at the genome-wide level using the following parameters as cutoffs: FC ≥ 2 and *P* < 0.05. In total, 3998 DEGs (1417 upregulated and 2581 downregulated) whose expression level was affected by treatment for 1 h at 37°C were identified (Figure 5A and Supplementary Table 1). Meanwhile, 1515 DEGs (533 upregulated and 982 downregulated) whose expression was differently regulated in 1-week-old *bin2-1* and Col-0 plants grown at 22°C under LD conditions were identified (Figure 5A and Supplementary Table 2). An examination of the RNA-Seq data revealed several genes among the 3998 HS-regulated DEGs whose expression was previously demonstrated to be upregulated by HS treatment, including *HSFA2*, *HSFA7a*, *HSFB2a*, *HSP90.1*, *HSP70*, *HSP60*, *HSP22*, *HSP21*, and *HSP18.2* (Li et al., 2019). Additionally, genes known to be downregulated by BR signaling, including *BR6OX2*, *CYP90D1*, *ROT3*, and *DWF4*, were found to be upregulated in the *bin2-1* mutant. Together, these results indicate that our HS treatment and RNA-Seq experiments were successful.

Among the DEGs identified in the *bin2-1* mutant, 608 (40.13%) were regulated by HS treatment (Figure 5B). These genes were designated HS and BIN2 coregulated DEGs. Interestingly, most of these HS and BIN2 coregulated DEGs (445, 73.19%) were downregulated by HS treatment and in the *bin2-1* mutant (Figure 5C and Supplementary Table 3). GO enrichment analysis showed that genes involved in plant hormone responses (e.g., to auxin, BR, ethylene, SA, JA, gibberellin, and ABA), defense responses (e.g., to chitin, wounding, the respiratory burst during defensive responses, and the immune effector process), and abiotic stress responses (e.g., to unfolded protein, light stimulus, osmotic stress, and heat stress) were enriched in 608 HS- and BIN2-coregulated DEGs (Figure 5D).

### BRASSINOSTEROID INSENSITIVE 2 Suppresses Plant Thermotolerance Partially Through ABSCISIC ACID INSENSITIVE 5

To uncover the transcription factors that mediate the expression of our HS- and BIN2-regulated DEGs, 1000 bp of genomic sequence, including the promoter and 5′-untranslated region located upstream of the ATG, from the 608 HS and BIN2 coregulated DEGs were subjected to local motif enrichment analysis using the MEME Suite webtool. *Cis*-elements that were recognized by AREB3, ABI5, GBF6, and/or ABF2 were the top-scoring motifs (Figure 5E). As the sequences of these enriched motifs are very similar and all contain an ACGT core sequence, and given that AREB3, ABI5, and ABF2 are important transcription factors in ABA signaling, HS and BIN2 likely regulate the expression of a subset of ABA-responsive genes to influence plant thermotolerance.

**FIGURE 6:**
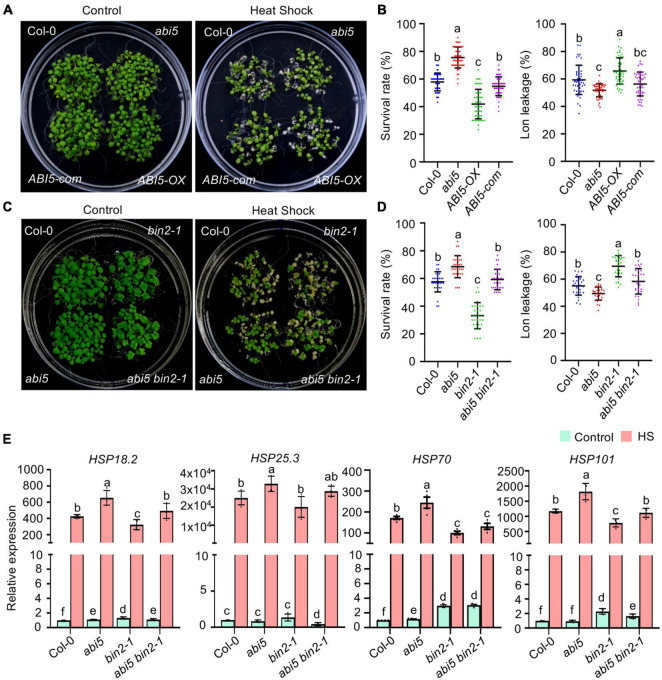
Abscisic acid (ABA) INSENSITIVE 5 (ABI5) functions downstream of BIN2 to regulate acquired thermotolerance in *Arabidopsis*. **(A,C)** Photographs of HS-treated and untreated plants after 3 days of recovery at 22°C. *ABI5-com* is an ABI5 complementation line in the *abi5* background. *ABI5-OX* was produced in the Col-0 background. **(B,D)** Quantification of the survival rates of and ion leakage from the plants shown in panels **(A,C)**. Error bars indicate the mean ± SD. Statistically significant differences are indicated by different lowercase letters (*p* < 0.05, one-way ANOVA). **(E)** Quantitation of *HSPs* transcript levels in one-week-old plants that were treated with 22°C (control) or 37°C (HS) for 1 h. Error bars indicate the mean ± SD (*n* = 4). Statistically significant differences are indicated by different lowercase letters (*p* < 0.05, Student’s *t*-test).

Previously, it was reported that *bin2-1* mutant plants were hypersensitive to ABA (Yang et al., 2016), and that BIN2 phosphorylates and stabilizes ABI5 to enhance the expression of ABI5-targeted genes (Hu and Yu, 2014). The enrichment of ABI5-recognizing *cis*-elements in our HS and BIN2 coregulated DEGs prompted us to test whether ABI5 participates in BIN2 regulated plant HS responses. We first examined if high temperature would regulate *ABI5* transcript or protein abundance. qPCR showed that 37°C treatment for 1 h could significantly increase the expression of *ABI5*. As HS treatment continues, the transcript level of *ABI5* starts to decrease and remain low or slightly lower than HS untreated plants (Supplementary Figure 2A). In comparison, ABI5-YFP protein from *ABI5pro:ABI5-YFP* transgenic plant remains unchanged when plants were subjected to 37°C for 2 h, but starts to accumulate continuously when HS treatment time increased from 4 h to 24 h (Supplementary Figure 2B). Next, we tested whether the expression level of *ABI5* would affect plant tolerance to HS treatment. Plants were exposed to 45°C for 60 min (non-acclimation conditions) or 3 h (acclimation conditions), and then allowed to recover at 22°C for 4 days. Quantification of the seedling survival rate showed that both the BT and AT were increased in the *abi5* mutant, which had no detectable ABI5 protein (Zhou et al., 2015), but decreased in transgenic plants overexpressing *ABI5* (*ABI5-OX*) (Hu and Yu, 2014). This enhanced thermotolerance seemed to be specifically regulated by the reduced expression of *ABI5* in the *abi5* mutant, as the transfer of the genomic *ABI5* sequence back into *abi5* (*ABI5 com*) plants restored the BT of *abi5* to a wild-type level, while it reduced the AT of *abi5* to a level that was lower than in the wild-type control (Figures 6A,B and Supplementary Figure 1I). Consistent with this, HS-induced electrolyte leakage was significantly lower in the *abi5* mutant, higher in *ABI5-OX* plants, and at a similar level in the *ABI5-com* plants compared with that in the wild-type control (Figure 6B). These results demonstrate that *ABI5* is a negative regulator of plant HS responses.

Because both *bin2-1* mutant and *ABI5-OX* plants were hypersensitive to HS treatment while BIN2 phosphorylation increased cellular ABI5 activity, we wondered whether increased ABI5 activity was responsible for the reduced thermotolerance of the *bin2-1* mutant. To test this hypothesis, we generated an *abi5 bin2-1* double mutant by genetic crossing and assessed its thermotolerance. Similar to the above results, both the BT and AT were significantly increased in the *abi5* mutant and decreased in *bin2-1* mutant plants. Introduction of the *abi5* T-DNA insertion mutation into a *bin2-1* mutant background increased the BT and AT of *bin2-1* plants to wild-type levels (Figures 6C,D and Supplementary Figure 1J). Together with our HS-induced electrolyte leakage data (Figure 6D), these results suggest that *bin2-1*-activated ABI5 partially contributes to the reduced thermotolerance observed in *bin2-1* plants.

Under stressful conditions, plants generally induce the expression of HSPs to prevent stress-induced protein aggregation and help ameliorate stress-induced cellular damage (Haq et al., 2019). To investigate whether BIN2 and ABI5 regulate a common set of downstream target genes to influence plant thermotolerance, the expression of *HSP18.2*, *HSP25.3*, *HSP70*, and *HSP101* was examined by qPCR in *bin2-1* and *abi5* plants. Treatment at 37°C for 1 h dramatically increased the expression of all four genes. In accordance with the thermotolerance of the mutants (Figures 6C,D and Supplementary Figure 1J), the expression of these *HSPs* was significantly increased in the *abi5* mutant, lower in the *bin2-1* mutant, and at a similar level for *HSP18.2*, *HSP25.3*, and *HSP101* in the *abi5 bin2-1* double mutant compared with that in Col-0 (control) plants (Figure 6E).

### Increased BRASSINOSTEROID INSENSITIVE 2 Activity Promotes Early Flowering

**FIGURE 7:**
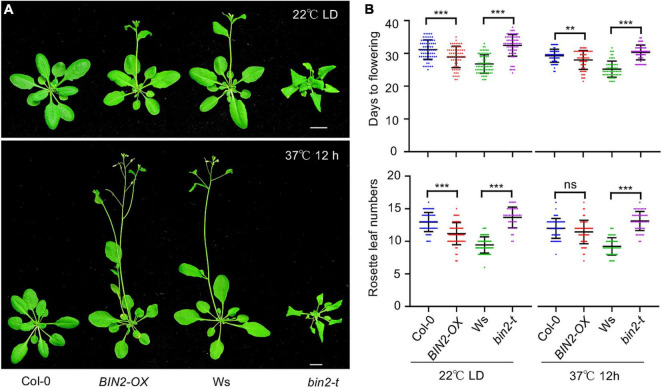
BRASSINOSTEROID INSENSITIVE 2 (BIN2) positively regulates flowering time in *Arabidopsis*. **(A)** Photographs of Col-0, *BIN2-OX*, Ws, and *bin2-3 bil1 bil2* (*bin2-t*) plants. Two-week-old plants grown in soil under LD conditions at 22°C were either exposed to 37°C for 12 h or kept at 22°C. All plants were then allowed to continue growing at 22°C until the first flower opened. **(B)** Quantitation of the days to flowering and the rosette leaf numbers for the flowering plants shown in panel **(A)**. Error bars indicate the mean ± SD (*n* ≥ 3). Statistical significance is indicated by asterisks (**p < 0.01; ***p < 0.001; ns, not significant, Student’s *t*-test).

The above results showed that HS induced the accumulation of BIN2, leading to ABI5 activation and reduced plant thermotolerance. We next considered how plants benefit from the HS-induced accumulation of BIN2. When *bin2-1* mutant, *bin2-t* mutant, and *BIN2-OX* plants were grown at 22°C under LD conditions, both the *bin2-1* mutant and *BIN2-OX* plants flowered 3–5 days earlier than Col-0 control plants (Figure 7 and Supplementary Figure 3), while the *bin2-t* mutant flowered about 5 days later than Ws control plants (Figure 7). Quantitation of the rosette leaves also showed that at the onset of flowering, the *bin2-1* mutant and *BIN2-OX* plants had fewer leaves than the Col-0 control (Figure 7 and Supplementary Figure 3), while the *bin2-t* mutant had more leaves than did the Ws control (Figure 7), indicating that increased BIN2 activity promotes early flowering in *Arabidopsis*.

One strategy that plants use to cope high temperatures is early flowering. If high temperatures really increase intracellular BIN2 activity to promote early flowering, the differences in flowering time between wild-type and *bin2-1* mutant or *BIN2-OX* plants should be smaller under high-temperature conditions. Accordingly, we heat-shocked 2-week-old plants (grown at 22°C under LD conditions) at 37°C for 12 h. The plants were then allowed to continue growing at 22°C under LD conditions until flowering. HS treatment promoted flowering in Col-0 (from 31 ± 3 days to 29 ± 2 days) and *BIN2-OX* (from 28 ± 3 days to 27 ± 3 days) plants such that it occurred 2 days and 1 day earlier, respectively. In addition, upon HS treatment, Col-0 had one less leaf (from 13 ± 1 to 12 ± 2) while *BIN2-OX* had the same number of leaves (from 11 ± 2 to 11 ± 2) when the plants started flowering. In comparison, following HS treatment, both the Ws (from 27 ± 3 days to 25 ± 2 days) and *bin2-t* mutant (32 ± 3 days to 30 ± 2 days) plants flowered 2 days earlier, and the Ws plants had the same number of rosette leaves (9 ± 1) while the *bin2-t* mutant (from 14 ± 2 to 13 ± 1) had one less leaf when the plants started flowering (Figure 7). Together, these results suggest that increased BIN2 activity contributes to early flowering under HS conditions.

## Discussion

Under high temperature stress, plants quickly adjust their cellular metabolism and gene expression patterns to cope with the changed environment. The exogenous application of plant phytohormones such as ABA, cytokinin, BRs, SA, or JA can mitigate heat-induced damage and increase the thermotolerance of plants (Li et al., 2021b). However, the mechanisms by which phytohormone signaling regulates plant thermotolerance are not well understood. In this study, by sequentially examining the thermotolerance of different BR signaling mutants and plants overexpressing major BR signaling components, we obtained comprehensive genetic evidence showing that BIN2 mediates the crosstalk between BR signaling and plant HS responses.

Several lines of evidence from our study suggest that HS can increase cellular BIN2 activity. First, HS induced the accumulation of BIN2 protein, while the *BIN2* transcript level was reduced. HS likely increases the *de novo* biosynthesis of BIN2 or enhances the protein’s stability, thereby increasing the cellular abundance of BIN2. Second, we recently showed that BZR1 phosphorylation by BIN2 and dephosphorylation by PP2A all happen in the nucleus (Wang et al., 2021). Mobilizing BZR1 into the nucleus by MG132 treatment increased the chance of an interaction between BZR1 and nuclear-localized PP2A and promoted BZR1 dephosphorylation, even in the absence of BRs (Wang et al., 2021). If the same principle applies to the HS-induced translocation of BZR1 to the nucleus, we should be able to observe an increased level of dephosphorylated BZR1 after HS treatment. The discovery that HS treatment could induce the nuclear localization of BZR1 while also decreasing the dephosphorylation level of BZR1 suggests that HS exposure increases BIN2 activity and prevents nuclear-localized BZR1 from being dephosphorylated by PP2A. Third, our transcriptomic analysis showed that HS exposure and the *bin2-1* mutation downregulate a similar group of genes that are involved in regulating plant responses to hormones and abiotic/biotic stresses. Taken together, these results suggest that HS suppresses plant thermotolerance by increasing BIN2 activity. Exogenously applied BRs promotes BIN2 degradation, thereby increasing plant thermotolerance.

Interestingly, 10 min of exposure to heat stress (37°C) quickly induced the nuclear translocation of BZR1, even in the absence of BRs. The HS-induced nuclear localization of BZR1 might be mediated by sumoylation because HS is known to induce massive protein sumoylation (Rytz et al., 2018) and the sumoylation of BZR1 promotes its nuclear localization (Srivastava et al., 2020). By RNA-Seq analysis, we found that BR metabolism- or biosynthesis-related genes were significantly downregulated by short-term (up to 30 min) exposure to 37°C. Also, a BZR1-recognizing *cis*-element was enriched in DEGs regulated by 10 min of HS treatment, suggesting that BZR1 helps regulate early plant responses to high temperature exposure (Li et al., 2019). It would be interesting to examine whether the accumulation of phosphorylated BZRs in the nucleus regulates the expression of downstream target genes via protein–protein interactions with other transcription factors.

As a protein kinase, BIN2 plays a major role in regulating plant growth and development and in plant responses to biotic/abiotic stresses via the phosphorylation of a variety of substrates (Mao et al., 2021). By motif analysis, we found that an ABI5-targeting *cis*-element was enriched in the 1-kb promoter sequences of HS and *bin2-1* coregulated DEGs. Similar to BIN2, HS treatment could also induce the accumulation of ABI5 protein. As ABA is a positive regulator of plant thermotolerance that increases the accumulation and activity of ABI5, it is surprising that the thermotolerance was increased in *abi5* but decreased in *ABI5-OX* plants. However, these data support findings showing that ABI5 is a BIN2 substrate and that BIN2 phosphorylation stabilizes and increases the activity of ABI5 (Hu and Yu, 2014). The increased thermotolerance of the *abi5 bin2-1* double mutant (compared with the *bin2-1* mutant) suggests that the HS-induced accumulation of BIN2 activates ABI5 to suppress plant thermotolerance. This conclusion can also explain the fact that the thermotolerance of the *bzr1-1D* mutant was similar to that of wild type, while the *bzr1-1D* mutation increased the thermotolerance of the *bin2-1* mutant. Previously, we showed that *ABI5* is a direct target of BZR1 and that bzr1-1D reduced the expression of *ABI5* in *bin2-1* mutant plants (Yang et al., 2016). This may help explain the partial reversal of the reduced thermotolerance in our *bin2-1* plants by *bzr1-1D* mutation. As both *bzr1-1D* and *abi5* could only partially recover the reduced thermotolerance of the *bin2-1* mutant, additional BIN2 substrates are probably also involved in regulating plant thermotolerance.

Early flowering is an adaptive trait that plants use to cope with biotic and abiotic stresses (Kazan and Lyons, 2016). In doing so, plants switch from vegetative to reproductive growth to ensure that the species can survive in an unfavorable environment. Though studies indicate that ambient high temperatures can promote early flowering by suppressing the expression of floral repressor genes such as *FLOWERING LOCUS C* (*FLC*), *FLOWERING LOCUS M* (*FLM*), and *SHORT VEGETATIVE PHASE* (*SVP*) through PIF4, FCA, and SPL3 (Capovilla et al., 2015), it is unknown whether a similar mechanism applies to heat-shocked plants. In this study, 12 h of treatment at 37°C during the day promoted early flowering in *Arabidopsis*. Increased BIN2 activity caused by the overexpression of *BIN2* or the *bin2-1* mutation promoted early flowering both at room temperature and after HS treatment. Conversely, the flowering time in the *bin2-t* mutant was significantly delayed, indicating that BIN2 is a positive regulator of flowering. However, when grown under LD conditions, the *abi5* mutant also flowered earlier, while *ABI5-OX* plants had a late-flowering phenotype (Wang et al., 2013), in contrast to the early flowering phenotypes of *bin2-1* and our *BIN2-OX* plants. Therefore, despite the fact that BIN2 suppresses plant thermotolerance by activating ABI5, ABI5 is not responsible for the early flowering observed in our *BIN2-OX* and *bin2-1* plants. BIN2 likely suppresses plant thermotolerance and promotes flowering via different downstream target proteins.

It is surprising that the *bin2-1* mutant and *BIN2-OX* plants flowered early because BR biosynthesis- and BR signaling-deficient mutants such as *det2*, *cpd*, *dwf4*, and *bri1* flower late (Azpiroz et al., 1998; Domagalska et al., 2007; Izawa, 2021). In contrast, the overexpression of BRI1 promotes early flowering (Singh et al., 2016). In the present study, under our growth conditions, *BIN2-OX* and *bin2-1* plants exhibited early flowering regardless of whether we counted the number of days to flowering or the number of rosette leaves at flowering. In the *bin2-1* mutant, the upstream BR signaling pathway is still intact while downstream BZRs are inactive. This could cause feedback-based upregulation of BR biosynthesis genes. Therefore, upstream BR signaling components should be hyperactive in the *bin2-1* mutant. Could upstream BR signaling components such as BRI1, BAK1, BSKs, and BSU1 promote flowering directly through a BIN2- and BZR-independent mechanism? This hypothesis could explain the severe late flowering phenotype of the *bzr1-1D* mutant; BR biosynthesis-related genes are downregulated in this mutant, and this would inactivate upstream BR signaling components. However, this hypothesis cannot explain why *BES1-OX* plants flowered early while *BES1*-RNAi plants flowered late (Wang et al., 2019). It is possible that BR signaling components regulate flowering time via multiple mechanisms.

In conclusion, by analyzing plants with mutations in or that overexpressed different BR signaling components, we found that BR signaling regulates plant thermotolerance by promoting BIN2 degradation. Additionally, our data suggest that to cope with HS exposure plants increase the abundance of BIN2 to promote flowering but at a cost of reducing plant thermotolerance. Together, our findings provide an excellent example of how plants sacrifice the survivability of individuals at high temperatures to ensure the survival of the species.

## Data Availability Statement

The original contributions presented in the study are publicly available. This data can be found here: The RNAseq datasets generated and analyzed for this study have been deposited in China National Center for Bioinformation (https://www.cncb.ac.cn/), with the project number: PRJCA007585.

## Author Contributions

WT and DS designed the research. HR, KG, and XW did the experiments with YW’s assistant and under the supervision of DS and WT. HR and WZ analyzed the RNA-seq data. KG and WT wrote the manuscript together. All authors contributed to the article and approved the submitted version.

## Conflict of Interest

The authors declare that the research was conducted in the absence of any commercial or financial relationships that could be construed as a potential conflict of interest.

## Publisher’s Note

All claims expressed in this article are solely those of the authors and do not necessarily represent those of their affiliated organizations, or those of the publisher, the editors and the reviewers. Any product that may be evaluated in this article, or claim that may be made by its manufacturer, is not guaranteed or endorsed by the publisher.

## Footnotes

1
https://www.ipcc.ch

2
www.biocloud.net

3
http://systemsbiology.cau.edu.cn/agriGOv2/

4
www.arabidopsis.org

5
https://meme-suite.org/meme/doc/centrimo.html?man_type=web

## Supplementary Material

Supplementary Figure 1 Brassinosteroids regulate plant basal thermotolerance by suppressing a BIN2-ABI5 module in *Arabidopsis*. **(A–J)** Survival rate of various BR biosyntheis or signaling mutants, and plants that overexpressing different BR signaling components. One-week-old seedlings grown on 1/2 MS agar medium supplied with or without 0.1 μM eBL under LD conditions at 22°C were grown at 45°C for 1 h. The survival rates of the plants were determined after 3 days of recovery at 22°C. Error bars indicate the mean ± SD. Statistically significant differences are indicated by different lowercase letters (*p* < 0.05, Student’s *t*-test).

Supplementary Figure 2 The regulation of ABI5 transcript and protein abundance by HS. **(A)** The relative expression of *ABI5* transcripts in 1-week-old Col-0 seedlings grown at 37°C for the indicated time. Error bars indicate the mean ± SD (*n* = 3). Statistically significant differences are indicated by different lowercase letters (*p* < 0.05, one-way ANOVA). **(B)** Immunoblotting revealed the abundance of ABI5-YFP in 1-week-old *ABI5pro:ABI5-YFP* expressing seedlings treated with 37°C for the indicated time.

Supplementary Figure 3 Increased cellular BIN2 activity or protein levels promote early flowering in *Arabidopsis*. **(A)** Phenotypes of Col-0, *bin2-1*, and *BIN2-OX* plants grown in soil at 22°C under LD conditions until the first flower opened. **(B)** Quantitation of the days to flowering and rosette leaf numbers for the flowering plants shown in panel **(A)**. Error bars indicate the mean ± SD. Different lowercase letters indicate statistically significant differences (*p* < 0.05, Student’s *t*-test).
