# Supplementary figures and images for: Heat Shock-Induced Accumulation of the Glycogen Synthase Kinase 3-Like Kinase BRASSINOSTEROID INSENSITIVE 2 Promotes Early Flowering but Reduces Thermotolerance in Arabidopsis
Source: Front Plant Sci. 2022 Jan 27;13:838062. doi: 10.3389/fpls.2022.838062 (PMC8828572; doi:10.3389/fpls.2022.838062)

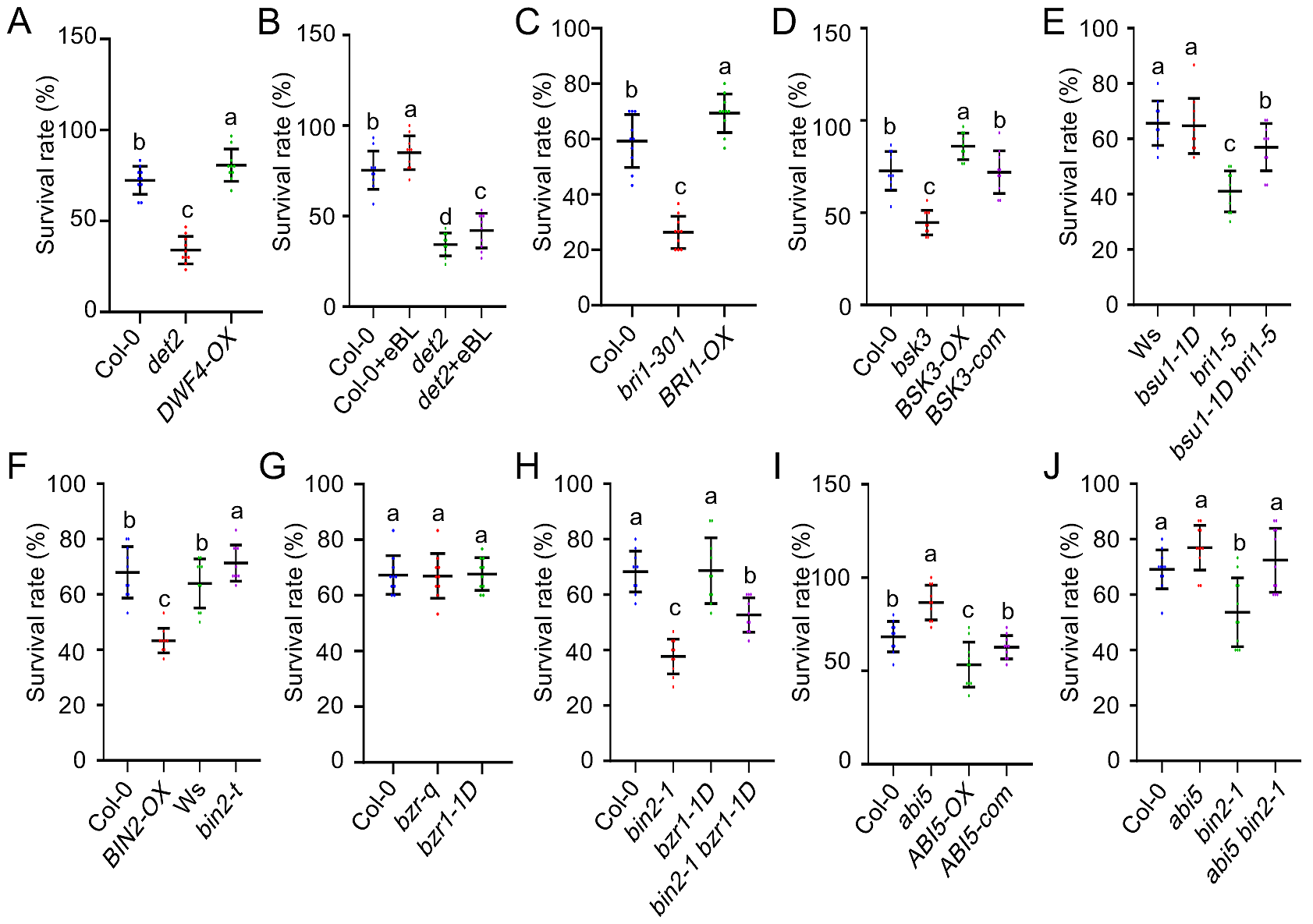

Supplement: Supplementary Figure 1 — Brassinosteroids regulate plant basal thermotolerance by suppressing a BIN2-ABI5 module in Arabidopsis. (A–J) Survival rate of various BR biosyntheis or signaling mutants, and plants that overexpressing different BR signaling components. One-week-old seedlings grown on 1/2 MS agar medium supplied with or without 0.1 μM eBL under LD conditions at 22°C were grown at 45°C for 1 h. The survival rates of the plants were determined after 3 days of recovery at 22°C. Error bars indicate the mean ± SD. Statistically significant differences are indicated by different lowercase letters (p < 0.05, Student’s t-test). [file Image_1.TIF]

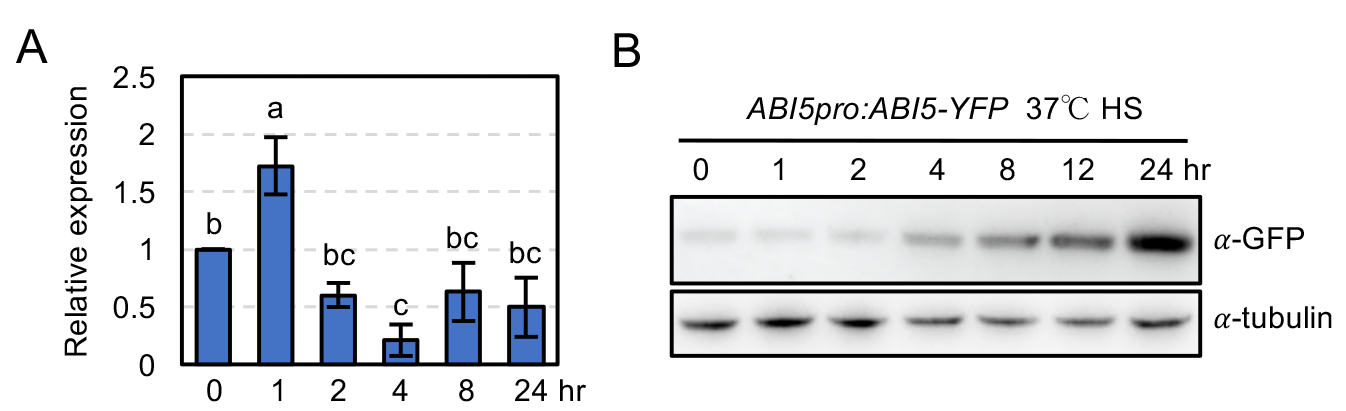

Supplement: Supplementary Figure 2 — The regulation of ABI5 transcript and protein abundance by HS. (A) The relative expression of ABI5 transcripts in 1-week-old Col-0 seedlings grown at 37°C for the indicated time. Error bars indicate the mean ± SD (n = 3). Statistically significant differences are indicated by different lowercase letters (p < 0.05, one-way ANOVA). (B) Immunoblotting revealed the abundance of ABI5-YFP in 1-week-old ABI5pro:ABI5-YFP expressing seedlings treated with 37°C for the indicated time. [file Image_2.TIF]

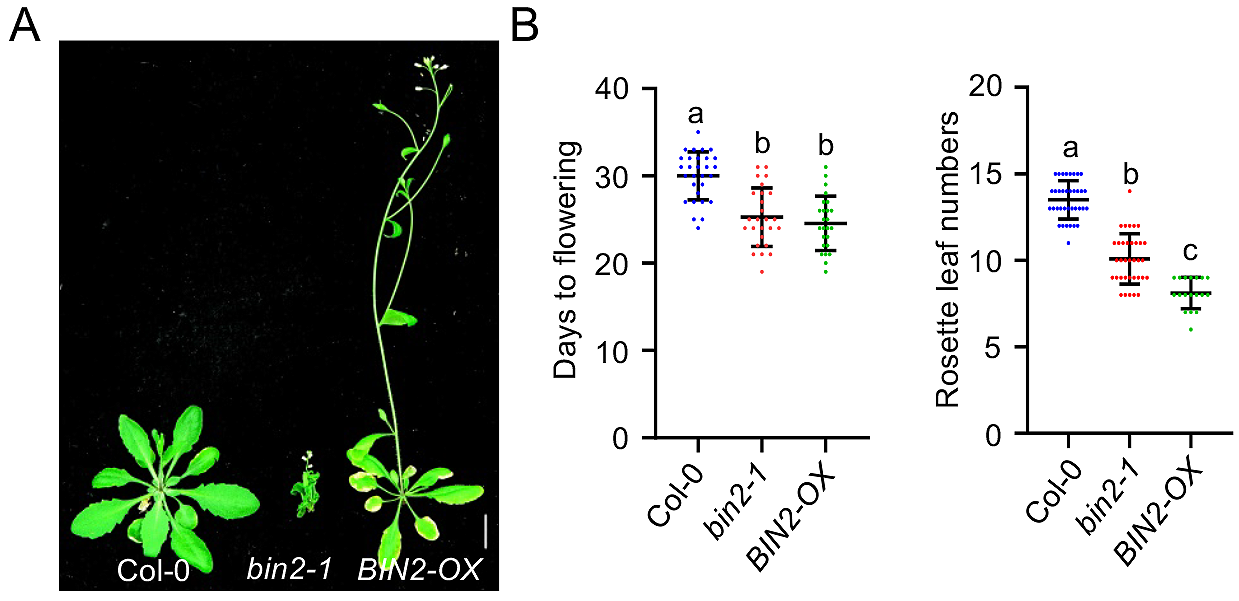

Supplement: Supplementary Figure 3 — Increased cellular BIN2 activity or protein levels promote early flowering in Arabidopsis. (A) Phenotypes of Col-0, bin2-1, and BIN2-OX plants grown in soil at 22°C under LD conditions until the first flower opened. (B) Quantitation of the days to flowering and rosette leaf numbers for the flowering plants shown in panel (A). Error bars indicate the mean ± SD. Different lowercase letters indicate statistically significant differences (p < 0.05, Student’s t-test). [file Image_3.TIF]
